# Proprioceptive Changes Impair Balance Control in Individuals with Chronic Obstructive Pulmonary Disease

**DOI:** 10.1371/journal.pone.0057949

**Published:** 2013-03-01

**Authors:** Lotte Janssens, Simon Brumagne, Alison K. McConnell, Kurt Claeys, Madelon Pijnenburg, Chris Burtin, Wim Janssens, Marc Decramer, Thierry Troosters

**Affiliations:** 1 Department of Rehabilitation Sciences, University of Leuven (KU Leuven), Leuven, Belgium; 2 Centre for Sports Medicine & Human Performance, Brunel University, Uxbridge, United Kingdom; 3 Department of Health Care, Catholic University College of Bruges-Ostend, Bruges, Belgium; 4 Respiratory Rehabilitation and Respiratory Division, University Hospitals Leuven, Leuven, Belgium; The University of Queensland, Australia

## Abstract

**Introduction:**

Balance deficits are identified as important risk factors for falling in individuals with chronic obstructive pulmonary disease (COPD). However, the specific use of proprioception, which is of primary importance during balance control, has not been studied in individuals with COPD. The objective was to determine the specific proprioceptive control strategy during postural balance in individuals with COPD and healthy controls, and to assess whether this was related to inspiratory muscle weakness.

**Methods:**

Center of pressure displacement was determined in 20 individuals with COPD and 20 age/gender-matched controls during upright stance on an unstable support surface without vision. Ankle and back muscle vibration were applied to evaluate the relative contribution of different proprioceptive signals used in postural control.

**Results:**

Individuals with COPD showed an increased anterior-posterior body sway during upright stance (p = 0.037). Compared to controls, individuals with COPD showed an increased posterior body sway during ankle muscle vibration (p = 0.047), decreased anterior body sway during back muscle vibration (p = 0.025), and increased posterior body sway during simultaneous ankle-muscle vibration (p = 0.002). Individuals with COPD with the weakest inspiratory muscles showed the greatest reliance on ankle muscle input when compared to the stronger individuals with COPD (p = 0.037).

**Conclusions:**

Individuals with COPD, especially those with inspiratory muscle weakness, increased their reliance on ankle muscle proprioceptive signals and decreased their reliance on back muscle proprioceptive signals during balance control, resulting in a decreased postural stability compared to healthy controls. These proprioceptive changes may be due to an impaired postural contribution of the inspiratory muscles to trunk stability. Further research is required to determine whether interventions such as proprioceptive training and inspiratory muscle training improve postural balance and reduce the fall risk in individuals with COPD.

## Introduction

Fall injuries in individuals with chronic obstructive pulmonary disease (COPD) are common [1]. A recent prospective study determined that approximately one third of the ambulatory patients with COPD report a fall during a period of six months. Moreover, a previous fall history seems to be the most important predictor of future fall risk in individuals with COPD [2]. Fall injuries in individuals with COPD are frequently associated with hip and vertebral fractures, which may be explained by the high prevalence of osteoporosis in COPD [3]. Furthermore, low levels of vitamin D are observed in individuals with COPD which impairs bone strength, muscle strength and balance, and thus in turn are a risk factor for osteoporosis and falls [4]. Accordingly, the consequences of falls in individuals with COPD may have major complications in terms of morbidity, mortality as well as costly use of healthcare services. The underlying mechanisms for the apparent association between COPD and fall risk remain to be examined systematically.

Balance deficits are identified as one of the intrinsic risk factors for falling in COPD, next to age, depression, malnutrition, cognitive impairments and medication intake [5], [6]. The effectiveness of human balance control depends on the availability, reliability and central processing of visual, vestibular and proprioceptive inputs and motor outputs [7]. Visual deficits are rarely reported in COPD, neither the association between visual deficits and falls in people with COPD [5]. Moreover, systematic vestibular problems seem unlikely as audio-vestibular dysfunctions have been observed to be uncommon in individuals with COPD [8]. However, the proprioceptive sense, which is of primary importance during postural control, has not been studied specifically in individuals with COPD.

Optimal upright standing requires proprioceptive control at the ankles, knees, hips and spine level, which is defined as multi-segmental control [9]. In the presence of pain, fatigue or injury, specific proprioceptive signals may lose reliability, and individuals may become more dependent on the remaining proprioceptive systems. For example, when back muscle proprioceptive signals lose reliability due to low back pain, these individuals adopt a more ankle-steered strategy to maintain balance, a process known as proprioceptive weighting [10]. When, subsequently, ankle proprioception becomes less reliable, for example while standing on unstable support surfaces [11], individuals with low back pain are less able to switch to a multi-segmental strategy, and this may strongly challenge their maintenance of balance [10]. The human diaphragm, which is mainly an inspiratory muscle, plays an important role in stabilizing the spine during balance and loading tasks [12]. It is reasonable to suggest that an increased demand for inspiratory function of the diaphragm might inhibit its trunk stabilizing contribution to balance. Therefore, we may speculate that the higher fall risk in COPD could be due to the effects of increased work of breathing upon the diaphragm contribution to postural control, which enforces reliance upon an ankle-steered control strategy. To evaluate this hypothesis, there is a need to determine the role of proprioception during balance control in individuals with COPD.

Some recent studies identified specific balance deficits in individuals with COPD. Individuals with COPD show an increased body sway in rest [13] and after an exercise task [14], [15], and have difficulty reaching beyond the length of their arms without losing balance [13], [16]. The balance deficits in individuals with COPD have been shown to impair dynamic task performance [13], [17] and responses to externally applied postural perturbations [2], [17]. The reasons for this remain unknown. Most studies have concluded that difficulties with balance are due to the limb muscle weakness in COPD [18]. However, the ability to generate a successful postural reaction following loss of balance is determined not only by motor output, but also by the effective use of proprioceptive inputs [9]. A comprehensive study characterizing proprioceptive postural control in COPD is lacking.

The main objective of this study was to compare the specific proprioceptive control strategy during postural balance between individuals with COPD and healthy controls, more specifically in relation to inspiratory muscle strength. We hypothesize that individuals with COPD, especially those with inspiratory muscle weakness, will show a maladaptive ankle-steered strategy during postural control, which may contribute to the impaired balance performance and consequently to the large fall risk in this population.

## Methods

### Ethics Statement

All study participants gave their written informed consent conform to the principles of the Declaration of Helsinki (1964). The study was approved by the local Ethics Committee of Biomedical Sciences, KU Leuven, Belgium (Clinical Trial Center: B322201112379-S53589) and registered at www.clinicaltrials.gov (NCT01505543).

### Subjects

Twenty individuals with COPD (6 women, 14 men) and 20 healthy controls participated voluntarily in this study. The two groups were matched for age (+/−2 years) and gender. Individuals with a history of specific balance problems (e.g. vestibular or neurological disorder), spinal surgery, or lower limb problems were excluded.

A physical activity questionnaire was completed [19]. Spirometry was evaluated using forced expiratory volume in one second (FEV_1_) and forced vital capacity (FVC). Respiratory muscle strength was evaluated by measuring maximal inspiratory pressure (PImax) and maximal expiratory pressure (PEmax) using an electronic pressure transducer (MicroRPM, Micromedical Ltd., Kent, UK). The PImax was measured at functional residual capacity and the PEmax at total lung capacity. A minimum of five repetitions was performed and tests were repeated until there was less than 5% difference between the best and second best test. The highest pressure sustained over 1 second was recorded and compared to reference values [20].

### Postural Stability and Proprioceptive Control Strategy

Postural sway characteristics were assessed by center of pressure (CoP) displacement using a 6-channel force plate (Bertec, OH, USA) which recorded the moment of force around the frontal axis (Mx), anterior-posterior force (Fy) and the vertical ground reaction force (Fz). Force plate signals were sampled at 500 Hz using a Micro1401 data acquisition system using Spike2 software (Cambridge Electronic Design, UK) and were filtered using a low pass filter with a cut-off frequency of 5 Hz.

Local muscle vibration was used to investigate the role of proprioception in postural control. Muscle vibration is a powerful stimulus of muscle spindle Ia afferents [21], [22]. It evokes an illusion of muscle lengthening in standing. If the central nervous system uses proprioceptive signals of the vibrated muscles for postural control, it will cause a directional corrective CoP displacement. When the tendon of the triceps surae is vibrated, a postural sway in a backward direction is expected, whereas during back muscle vibration, a forward postural body sway is expected, which has been shown by previous studies [10], [23]. The amount of CoP displacement represents the extent to which an individual makes use of the proprioceptive signals of the vibrated muscles to maintain the upright posture. Muscle vibrators (Maxon motors, Switzerland) were applied bilaterally over the most proximal part of the tendon of the triceps surae muscles and over the lumbar paraspinal muscles at the level of L5, and vibration was offered at a high frequency and low amplitude (60 Hz, 0.5 mm) [21].

### Study Design

**Figure 1:**
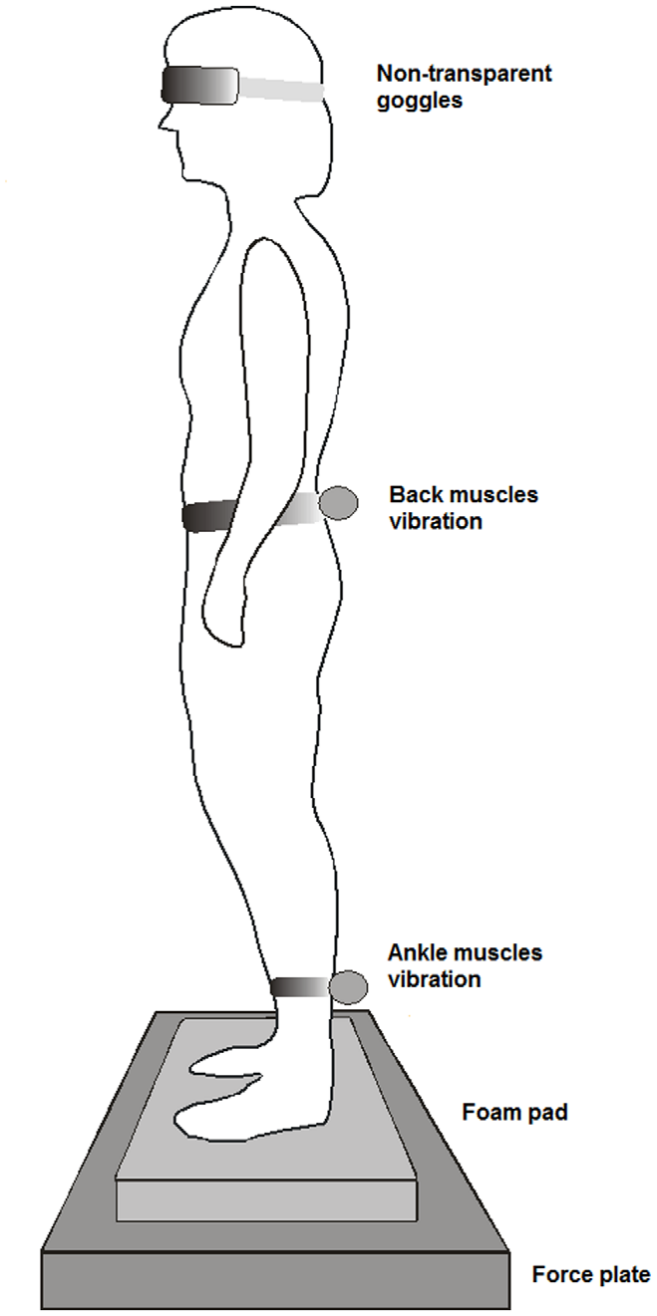
Experimental set-up. Standing on unstable support surface on force plate with ankle and back muscles vibration and vision occlusion.

Figure 1 shows the experimental set-up. The participants were instructed to stand barefoot on a foam pad (Airex balance pad; 49.5 cm length×40.5 cm width×6.5 cm height), placed on the force plate. On unstable support surface, ankle proprioceptive signals are less reliable, which enforces reliance upon proximal proprioceptive signals (i.e., proprioceptive weighting), thereby highlighting proprioceptive deficits [11]. A standardized foot position was used, with the heels placed 10 centimeter apart, and a free forefoot position. The vision of the participants was occluded by means of non-transparent goggles. Participants were instructed to maintain their balance at all times and an investigator was standing next to the participant to prevent actual falls. Four experimental trials were implemented (Table 1). Postural stability was measured in trial 1 by standing on unstable support surface without vision for 30 seconds. Subsequently, muscle vibration was added bilaterally for 15 seconds to the ankle muscles (trial 2), back muscles (trial 3), and to the ankle and back muscles simultaneously (trial 4). Each trial was performed once. Between trials, one minute of rest was taken and the subjects were asked to move their lower limbs and pelvis briefly to reset muscle spindles.

**Table 1:** Experimental trials.

Trial	Description
**1**	Upright stance on unstable support surface without vision (30 sec)
**2**	Upright stance on unstable support surface without vision (15 sec) – bilateral **ankle** muscles vibration (15 sec)
**3**	Upright stance on unstable support surface without vision (15 sec) – bilateral **back** muscles vibration (15 sec)
**4**	Upright stance on unstable support surface without vision (15 sec) – bilateral **ankle and** **back** muscles vibration (15 sec)

### Data Reduction and Statistical Analysis

Force plate data were calculated using Spike2 software and Microsoft Excel. Root Mean Square (RMS) values of the CoP displacements were used for the analysis of postural stability (Trial 1). To evaluate the directional effect of muscle vibration (Trial 2–4), mean values of anterior-posterior CoP displacement were calculated by using the equation: CoP =  (-h*Fy+Mx)/Fz) with *h* as the foam pad thickness (6.5 cm) and *Fy* as the anterior-posterior force. Positive values indicate a forward body sway and negative values indicate a backward body sway. To provide additional information about the proprioceptive dominance, a Relative Proprioceptive Weighting ratio (RPW) was calculated using the equation: RPW =  (Abs ankle)/(Abs ankle+Abs back). ‘Abs ankle’ is the absolute value of the mean CoP displacement during ankle muscle vibration and ‘Abs back’ during back muscle vibration. A RPW score equal to one corresponds to 100% reliance on ankle muscle input (‘ankle-steered strategy’), whereas a score equal to zero corresponds to 100% reliance on back muscle input (‘multi-segmental strategy’) [10], [23].

A one-way analysis of variance (ANOVA) was used to examine differences in baseline characteristics between the two groups (Table 1). A repeated measures ANOVA was used to examine differences between subjects and within-subjects. A post hoc test (Tukey) was performed to further analyze these results in detail. A mean split analysis was used to subgroup the individuals with COPD. The statistical analysis was performed with Statistica 9.0 (Statsoft, USA). The level of significance was set at p<0.05.

## Results

### Subject Characteristics

Two individuals with COPD, and therefore also 2 controls, were excluded from data analysis since they could not maintain their balance without manual assistance. The characteristics of both groups are displayed in Table 2. The individuals in the COPD group had a known diagnosis of COPD based on the GOLD criteria [24]. They had stable COPD and were distributed over GOLD stages II-IV. None of the healthy participants had a history of smoking or evidence of airflow obstruction. Respiratory muscle strength was significantly lower in the COPD group compared to the control group. The individuals with COPD were subdivided based on their PImax performance, placing those with a PImax<mean PImax (85±23% predicted) in a subgroup characterized by inspiratory muscle weakness and those with a PImax>mean PImax in a subgroup without inspiratory muscle weakness.

**Table 2:** Participant characteristics.

	Control group(n = 18)			COPD group(n = 18)			p-value
**Age (yrs)**	64	±	6	64	±	7	0.881
**Height (cm)**	173	±	9	169	±	7	0.213
**Weight (kg)**	74	±	11	75	±	14	0.821
**BMI (kg/m^2^)**	25	±	3	26	±	4	0.313
**PAI**	9.1	±	1.3	8.3	±	1.1	0.126
**FVC (% pred)**	117	±	13	91	±	24	**0.001**
**FEV_1_ (% pred)**	110	±	16	50	±	18	**0.001**
**FEV_1_/FVC**	75	±	9	45	±	13	**0.001**
**FRC (% pred)**	N/A			145	±	37	**N/A**
**PImax (% pred)**	112	±	24	85	±	23	**0.002**
**PEmax (% pred)**	120	±	24	101	±	31	**0.045**

Data are presented as mean ± standard deviation. BMI: Body Mass Index; PAI: Physical Activity Index (maximum score = 15); FVC: Forced Vital Capacity; FEV_1_: Forced Expiratory Volume in 1 second; FRC: Functional Residual Capacity; PImax: maximal inspiratory pressure; PEmax: maximal expiratory pressure; % pred: percentage predicted; N/A: not applicable; Significant p-values (p<0.05) in bold.

### Postural Stability

**Figure 2:**
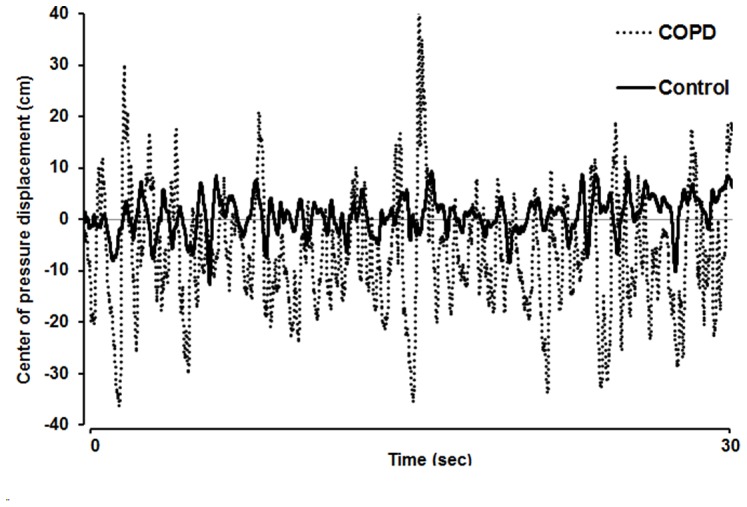
Postural stability. Raw data of center of pressure (CoP) displacement of an individual with COPD and an age/gender-matched healthy individual while standing on unstable support surface without vision for 30 seconds. Positive values indicate an anterior body sway, negative values indicate a posterior body sway.

During upright stance on unstable support surface, individuals with COPD showed an increased body sway in anterior-posterior direction (RMS: 6.5±3.0 cm) when compared to controls (RMS: 4.5±1.5 cm) (p = 0.037). Figure 2 displays representative raw data.

### Proprioceptive Control Strategy

**Figure 3:**
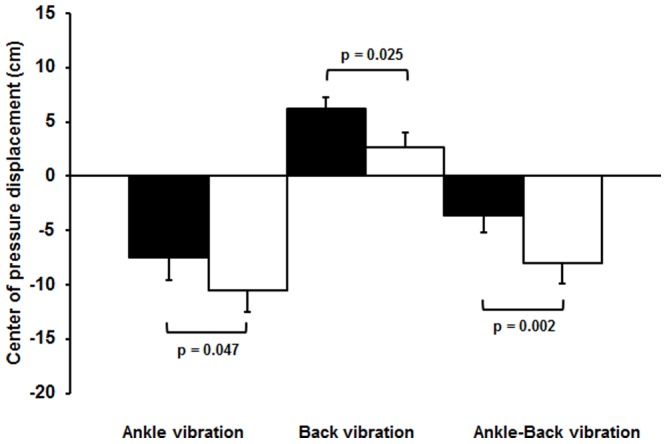
Proprioceptive control strategy. Center of pressure displacement (mean ± SD) in the control group (black) and COPD group (white) during vibration on ankle muscles, back muscles, and simultaneously on ankle and back muscles. Positive values indicate an anterior body sway, negative values indicate a posterior body sway.

Figure 3 compares proprioceptive postural control for all vibration trials. Individuals with COPD showed an increased reliance on ankle proprioceptive signals during postural control as shown by a larger posterior body sway (−10.4±4.1 cm) during ankle muscle vibration compared to controls (−7.6±4.4 cm) (p = 0.047). This was corroborated by the finding that the COPD group showed a lower reliance on back muscle signals to maintain balance, as anterior body sway (3.0±2.4 cm) during back muscle vibration was reduced compared to controls (6.2±2.2 cm) (p = 0.025). Simultaneous ankle-back muscle vibration elicited significantly larger posterior body sways in individuals with COPD (−7.6±3.7 cm) compared to controls (−3.8±3.2 cm) (p = 0.002), indicative of a dominant use of ankle proprioceptive signals during postural control.

**Figure 4:**
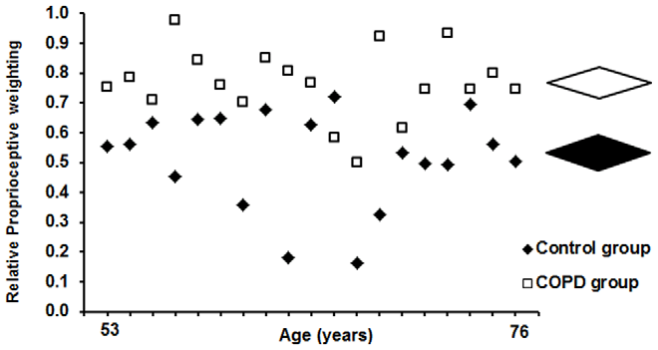
Relative proprioceptive weighting. Individual (left) and mean ± SD (right) relative proprioceptive weighting (RPW) ratios in the COPD group (white) and age/gender-matched control group (black). Higher values correspond to higher reliance on ankle muscle proprioception; lower values correspond to higher reliance on back muscle proprioception.

Figure 4 displays the individual RPW ratios of the COPD and control group. The RPW ratios confirmed that individuals with COPD showed a more ankle-steered postural control strategy (RPW ratio: 0.77±0.12) than controls, who showed a more back-steered, multi-segmental postural control strategy (RPW ratio: 0.52±0.16) (p = 0.001). With the exception of one participant, all individuals with COPD demonstrated higher RPW ratios, and thus a suboptimal ankle-steered postural control strategy when compared to the controls.

### Proprioceptive Control Strategy in Relation to Inspiratory Muscle Strength

Table 3 displays the mean CoP displacements during ankle, back and ankle-back muscle vibration, and the RPW ratios of both subgroups (based on PImax) of individuals with COPD. Anthropometrics, age and spirometry did not differ between the two subgroups (p>0.05). The subgroup with the weakest inspiratory muscles exhibited the greatest reliance upon ankle proprioceptive signals compared to the subgroup with the stronger inspiratory muscles (p = 0.037). There was also a trend suggesting lower reliance upon back muscle proprioception in the subgroup with the weakest inspiratory muscles (p = 0.055). Based on the RPW ratios, the weaker subgroup clearly displayed a more ankle-steered strategy during postural control compared to individuals with COPD without inspiratory muscle weakness (p = 0.013).

**Table 3:** Proprioceptive control strategy in individuals with COPD in relation to inspiratory muscle strength.

Trial	COPD group						
	PImax >85% (n = 9)			PImax <85% (n = 9)			p-value
**CoP displacement during ankle muscle vibration (cm)**	−8.7	±	2.2	−11.2	±	3.3	**0.037**
**CoP displacement during back muscle vibration (cm)**	3.9	±	2.6	2.0	±	2.0	0.055
**CoP displacement during ankle-back muscle vibration (cm)**	−6.1	±	3.6	−8.1	±	2.8	**0.037**
**RPW**	0.71	±	0.11	0.81	±	0.11	**0.013**

Data are presented as mean ± standard deviation. COPD: chronic obstructive pulmonary disease; PImax: maximal inspiratory pressure; CoP: center of pressure; RPW: relative proprioceptive weighting ratio; cm: centimeter; Significant p-values (p<0.05) in bold.

## Discussion

Individuals with COPD exhibit an increased reliance on ankle muscle proprioceptive signals and a decreased reliance on back muscle proprioceptive signals while maintaining postural balance. As a result, they utilize an ankle-steered postural control strategy, making them vulnerable to falls when active on an unstable support surface (e.g., on sand), where ankle proprioceptive signals become less reliable. This may explain the decreased postural stability observed in individuals with COPD. Interestingly, individuals with COPD with the weakest inspiratory muscles show the greatest reliance on ankle muscle input when compared to those with better preserved inspiratory muscle function. These novel findings shed some light on possible sensory mechanisms contributing to the suboptimal balance control in individuals with COPD, and their greater fall risk.

To our knowledge, this study is the first to evaluate the underlying proprioceptive changes in balance control in individuals with COPD. Recently, Beauchamp et al. revealed that balance deficits in individuals with COPD are most prominent when performing tasks that require anticipatory postural adjustments [17]. The latter have been shown previously to involve feed-forward activation of the diaphragm [12]. These findings are consistent with our results, as well as with the notion that the role of the respiratory muscles in balance performance in COPD is impaired. Taken together, these data suggest that balance is not only determined by motor output, but also by the contribution and central processing of sensory inputs from those muscles [25]. When the function of these muscles is impaired, there appears to be a deficit in both the motor and sensory contributions to balance.

The dual role of the respiratory muscles must be considered in this context [12]. When the respiratory function of the diaphragm is challenged, e.g. when respiratory demand increases, its postural function may also be challenged, resulting in a negative effect upon postural control [26], [27]. Healthy individuals are able to compensate for increases in respiratory demand using multi-segmental control [28]. However, this compensation seems to be impaired when the demand for inspiratory muscle work increases, e.g. after inspiratory muscle loading [23]. The present study demonstrated that individuals with COPD show poor proprioceptive control, particularly when inspiratory muscle strength is decreased. Our results confirmed that individuals with COPD have inspiratory muscle weakness [29]. Our novel finding was that the weakest individuals with COPD exhibited the greatest reliance upon ankle proprioceptive signals, suggesting that inspiratory muscle weakness contributes to impaired proprioceptive postural control. Therefore, it is tempting to speculate that the use of a maladaptive ankle-steered strategy in COPD is due to the inability of the respiratory musculature to make its normal contribution to postural control.

Another explanation for the altered postural control strategy associated with COPD may be found in their hyperinflation, which also induces functional weakening of the inspiratory muscles. Airway obstruction and loss of lung elasticity lead to premature airway collapse and an increase in end-expiratory lung volume. Hyperinflation forces the sternum more anteriorly and causes a loss in thoracolumbar spine mobility [30]. Kantor et al. demonstrated that the postural compensation to respiratory perturbation is dependent upon postural chain mobility. The free play of joints associated in postural control seems essential to control balance in an effective multi-segmental way [31], especially when breathing volume is increased [32]. Thus, loss of spinal mobility may contribute to the use of a more rigid ankle-steered postural control strategy in individuals with COPD. Since hyperinflation induces both loss of spinal mobility, and functional inspiratory muscle weakening, this mechanism is also consistent with the apparent interrelationship of postural control and inspiratory muscle weakness.

The proprioceptive changes in individuals with COPD might also be attributed to the adverse side effects of frequently prescribed medications in the management of COPD. The detrimental effects of corticosteroids and psychotropics include dizziness, visuals deficits and both respiratory and peripheral muscle dysfunction [5]. In the presence of dizziness or altered vision, individuals are enforced to increase their reliance upon proprioceptive signals to maintain balance (i.e. sensory reweighting) [7], a process which might highlight proprioceptive deficits and thus may induce the use of an ankle-steered proprioceptive strategy in individuals with COPD. Moreover, we may speculate that the harmful effect of medication on respiratory muscle strength might in turn negatively affects the trunk stabilizing function of the diaphragm [12]. Given the indispensable medication intake of individuals with COPD, the possible side effects may contribute to the maladaptive proprioceptive strategies in individuals with COPD.

The present findings suggest that maladaptive proprioceptive strategies are an important risk factor for balance deficits among individuals with COPD. However, further studies are required to assess the relation with fall incidence, to evaluate proprioceptive strategies during dynamic postural tasks, and to explore alternative factors (e.g. reduced plantar cutaneous sensation) which may explain balance deficits in individuals with COPD. Furthermore, our data justify further research using additional three-dimensional motion analysis may further unravel the altered proprioceptive strategies observed in individuals with COPD. The finding of a relationship between maladaptive ankle-steered postural control strategies and inspiratory muscle weakness justifies an interventional study to evaluate whether this is coincidental, or part of the underlying mechanism for poor balance control in individuals with COPD.

The results of our study support the idea that balance training should be implemented in the rehabilitation of COPD. Current balance training programs focus mainly on the balance performance in terms of ‘keeping or not keeping’ balance on unstable support surfaces. However, our findings suggest that a specific training approach, such as proprioceptive training, will be necessary to achieve an optimal multi-segmental postural strategy in individuals with COPD.

### Conclusions

Individuals with COPD, especially those with inspiratory muscle weakness, increased their reliance on ankle muscle proprioceptive signals and decreased the reliance on back muscle proprioceptive signals during balance control, resulting in a decreased postural stability compared to healthy controls. This maladaptive ankle-steered strategy in individuals with COPD might be explained by an impaired postural contribution of the inspiratory muscles to trunk stability. These findings provide a potential risk factor for fall incidence among this population, but also open a window of opportunity for intervention studies. Proprioceptive training and inspiratory muscles training warrant further study in the prevention of falls in individuals with COPD and their major consequences.
